# Effects of Fluoxetine and Visual Experience on Glutamatergic and GABAergic Synaptic Proteins in Adult Rat Visual Cortex^1^,^2^,^3^

**DOI:** 10.1523/ENEURO.0126-15.2015

**Published:** 2016-01-04

**Authors:** Simon Beshara, Brett R. Beston, Joshua G. A. Pinto, Kathryn M. Murphy

**Affiliations:** 1 McMaster Integrative Neuroscience Discovery and Study (MiNDS) Program, McMaster University, Hamilton, Ontario L8S 4K1, Canada; 2 Department of Psychology, Neuroscience & Behavior, McMaster University, Hamilton, Ontario L8S 4K1, Canada; 3 Department of Psychology, University of Toronto Mississauga, Mississauga, L5L 1C6, ON; 4 Health Care Investment Banking, Credit Suisse AG, San Francisco, CA 94108

**Keywords:** adult plasticity, amblyopia, fluoxetine, monocular deprivation, receptors, visual cortex

## Abstract

Fluoxetine has emerged as a novel treatment for persistent amblyopia because in adult animals it reinstates critical period-like ocular dominance plasticity and promotes recovery of visual acuity. Translation of these results from animal models to the clinic, however, has been challenging because of the lack of understanding of how this selective serotonin reuptake inhibitor affects glutamatergic and GABAergic synaptic mechanisms that are essential for experience-dependent plasticity. An appealing hypothesis is that fluoxetine recreates a critical period (CP)-like state by shifting synaptic mechanisms to be more juvenile. To test this we studied the effect of fluoxetine treatment in adult rats, alone or in combination with visual deprivation [monocular deprivation (MD)], on a set of highly conserved presynaptic and postsynaptic proteins (synapsin, synaptophysin, VGLUT1, VGAT, PSD-95, gephyrin, GluN1, GluA2, GluN2B, GluN2A, GABA_A_α1, GABA_A_α3). We did not find evidence that fluoxetine shifted the protein amounts or balances to a CP-like state. Instead, it drove the balances in favor of the more mature subunits (GluN2A, GABA_A_α1). In addition, when fluoxetine was paired with MD it created a neuroprotective-like environment by normalizing the glutamatergic gain found in adult MDs. Together, our results suggest that fluoxetine treatment creates a novel synaptic environment dominated by GluN2A- and GABA_A_α1-dependent plasticity.

## Significance Statement

Patching therapy is the most common treatment for children with amblyopia. For many, the acuity recovered during patching is lost when the treatment stops leaving the child with persistent amblyopia. Fluoxetine has emerged as an interesting treatment option because it reinstates critical period-like ocular dominance plasticity and promotes acuity recovery in adult animals. It remains unclear, however, how this selective serotonin reuptake inhibitor affects visual cortex plasticity, which relies heavily on glutamatergic and GABAergic synapses. Here we report the effects of fluoxetine and visual manipulation on the visual cortex of adult rats. Surprisingly we found that fluoxetine did not reinstate a critical period-like state, but rather created a novel synaptic environment that favors mature NMDA and GABA_A_ receptor subunits.

## Introduction

Amblyopia is commonly treated with patching, but in some cases, the recovered acuity is lost when patching stops, leaving the child with persistent amblyopia (Birch, 2013). A variety of therapeutics have been proposed to treat persistent amblyopia in adolescents or young adults. Fluoxetine has emerged as a treatment option because it reinstates critical period (CP)-like ocular dominance plasticity and promotes acuity recovery in adult rats (Vetencourt et al., 2008). It is unclear, however, what effects this selective serotonin reuptake inhibitor has on visual cortex (V1) plasticity that relies heavily on maturation of glutamatergic and GABAergic synapses (Levelt and Hübener, 2012). An appealing idea is that fluoxetine shifts the synaptic environment in V1 to a CP-like state that supports heightened experience-dependent plasticity.

During the CP, experience-dependent plasticity is driven by visually evoked responses that depend upon maturation of presynaptic and postsynaptic mechanisms. Development of presynaptic vesicle cycling proteins (eg, synapsin, synaptophysin) and transporters (eg, VGLUT, VGAT) are necessary for reliable neurotransmitter release (Hopf et al., 2002; Conti et al., 2004; Wojcik et al., 2004, 2006) that drives strong visually evoked activity. Also, shifts in the excitation/inhibition (E/I) balance set up the physiological environment needed for heightened plasticity, triggering the CP (Hensch, 2005; Hensch and Fagiolini, 2005). That E/I balance is mediate by postsynaptic scaffolding proteins PSD-95 and gephyrin that regulate the number of excitatory and inhibitory synapses (Prange et al., 2004; Lardi-Studler et al., 2007; Keith and El-Husseini, 2008). Furthermore, the start of the CP in rat and human V1 coincides with a rapid switch from much more gephyrin to an equal balance with PSD-95 (Pinto et al., 2013, 2015).

A host of glutamatergic and GABAergic receptor mechanisms affect the threshold for CP plasticity. These include addition of AMPA receptors (AMPARs) that end the period of NMDA receptor (NMDAR)-dominated silent synapses (Huang et al., 2015) and add the fast component to EPSCs (Kleppe and Robinson, 1999). Furthermore, the addition of GluN2A-containing NMDARs (Flint et al., 1997; Stocca and Vicini, 1998) speeds up receptor kinetics (Cull-Candy et al., 2001) and affects signaling pathways, such as GluN2B activation of Ras/ERK or alpha calcium-calmodulin kinase II and mTOR pathways (Kim et al., 2005; Wang et al., 2011). The shift to GluN2A also affects functional maturation by mediating sharpening of orientation selectivity (Fagiolini et al., 2003). Finally, activation of GABA_A_ receptors (GABA_A_Rs) triggers the start of the CP (Hensch, 2005), and GABA_A_α1 subunits in particular regulate patterns of activity needed for development of ocular dominance (Fagiolini et al., 2004).

Despite our understanding of the influence of fluoxetine treatment on adult plasticity, there is little evidence to identify “how” fluoxetine affects the expression profile of synaptic mechanism that are critical in the initiation of CP plasticity. To address this, we treated animals with fluoxetine and quantified a set of glutamatergic and GABAergic synaptic proteins to assess if they changed to a CP-like state. We then determined the effects of monocular deprivation (MD) alone, or in combination with fluoxetine on these synaptic proteins. Surprisingly, fluoxetine alone shifted both NMDAR and GABA_A_R subunits to a more mature composition. Furthermore, when fluoxetine was combined with MD, the treatment normalized the increase in glutamatergic proteins found in adult MD rats. These results show that fluoxetine treatment does not recreate a CP-like synaptic environment but instead shifts plasticity mechanisms to a new state.

## Materials and Methods

### Animals and surgical procedures

We studied changes in expression of 12 synaptic proteins in V1 of young adult male Long–Evans rats (P98). Rats were individually housed in Plexiglas cages with food and water *ad libitum*, and a 12 h light/dark cycle. Animals were randomly assigned to one of four groups: normally reared to P98 (*n* = 6), animals given 4 weeks of fluoxetine (from P70 to P98; 0.2 mg/ml of drinking water; *n* = 8), animals monocularly deprived (MDed; P91–P98; *n*=6), or animals that received both fluoxetine (P70–P98) and MD (P91–P98; n=8). Eyelids were closed by trimming the lid margins and suturing them together with 5-0 vicryl using aseptic surgical techniques. The surgery was done using gaseous anesthetic [isoflurane (1.5–5%) in oxygen] for induction and maintenance of anesthesia. Eyelids were inspected daily for openings. All experimental procedures were approved by the [McMaster University] Animal Research Ethics Board.

### Tissue collection

Animals were euthanized (sodium pentobarbital, 150 mg/kg), and transcardially perfused with cold 0.1 m PBS (4°C; 4–5 ml/min) until circulating fluid was clear. The brain was quickly removed from the skull and immersed in cold PBS. Bilateral samples of V1 including monocular and binocular regions, quickly frozen on dry ice, and stored at −80°C.

### Sample preparation

To study high-abundance vesicle cycling proteins and receptor scaffolding proteins (synapsin, synaptophysin, PSD-95, gephyrin) we prepared homogenate samples. The frozen tissue was added to cold homogenization buffer (1 ml buffer:50 mg tissue containing the following: 0.5 mm DTT, 1mm EDTA, 2 mm EGTA, 10 mm HEPES, 10 mg/L leupeptin, 100 nm microcystin, 0.1 mm PMSF, 50 mg/L soybean trypsin inhibitor) and homogenized in a glass–glass Dounce homogenizer (Kontes). The sample was then combined with 10% SDS. To study lower abundance receptor subunits (GluA2, GluN1, GluN2A, GluN2B, GABA_A_α1, GABA_A_α3) and transporters (VGLUT1, VGAT), we enriched the samples following a synaptoneurosomes protocol (Hollingsworth et al., 1985; Quinlan et al., 1999; Murphy et al., 2014). Following the homogenization step each sample was passed through a 5 μm pore hydrophilic mesh filter (Millipore), then centrifuged at 1000 × *g* for 10 min. Both the synaptic pellet and the whole-homogenate samples were resuspended in boiling 1% SDS. Protein concentrations for each sample was determined using the bicinchoninic acid assay guidelines (Pierce) and ﬁnal concentrations were adjusted to 1mg/ml using Laemmli sample buffer. A control sample was made by combining a small amount of each of the 28 samples.

### Immunoblotting

Samples (25 μg) were resolved on 4–20% SDS–PAGE gels (Precise Protein Gels, Pierce Biotechnology) and transferred to polyvinylidene difluoride (PVDF-FL) membranes (Millipore). Blots were incubated with blocking buffer (Odyssey Blocking Buffer 1:1 with PBS) for 1 h (LI-COR Biosciences), then with primary antibody overnight at 4°C using the following concentrations: GAPDH, 1:4000 (Imgenex); synapsin 1, 1:8000 (Invitrogen); synaptophysin, 1:2000 (Sigma-Aldrich); PSD-95, 1:32000 (Millipore); gephyrin, 1:2000 (Millipore); VGLUT1, 1:2000 (Synaptic Systems); VGAT, 1:2000 (Synaptic Systems); GluA2, 1:2000 (Invitrogen); GluN1, 1:8000 (Chemicon International); GluN2B, 1:2000 (Chemicon International); GluN2A, 1:2000 (PhosphoSolutions); GABA_A_α1 1:500 (Santa Cruz Biotechnology); GABA_A_α3 1:2000 (Chemicon International). The blots were washed with PBS containing 0.05% Tween (Sigma-Aldrich; PBS-T; 3 × 10 min), incubated for 1 h at room temperature with the appropriate IRDye-labeled secondary antibody, (anti-mouse, 1:8000; anti-rabbit, 1:10,000; LI-COR Biosciences), and washed in PBS-T (3 × 10 min). The blots were visualized using an Odyssey scanner (LI-COR Biosciences). The combination of IRDye secondary antibodies and Odyssey scanner provides a wide linear dynamic range so that both strong and weak bands could be accurately quantified on the same blot. Blots were stripped and reprobed with additional antibodies (Blot Restore Membrane Rejuvenation kit, Millipore).

### Analyses

To analyze the bands, we scanned the blots (Odyssey infrared scanner) and quantified the bands using densitometry (LI-COR Odyssey Software v3.0; LI-COR Biosciences). Density profiles were determined by performing a subtraction of the background, integrating the pixel intensity across the area of the band, and dividing the intensity by the width of the band to control for variations in lane width. Protein loading was checked using GAPDH as a control for sample concentration and volume loaded in each well. Each band was normalized to the average for the set of blots run at the same time and the control sample on the individual blot.

To quantify the relationship between functional pairs of proteins we calculated contrast indices that are commonly used in signal processing to determine the quality of the signal. AMPAR-NMDAR index: (GluA2-GluN1)/(GluA2+GluN1). NMDAR index: (GluN2A-GluN2B)/(GluN2B+GluN2A). GABA_A_R Index – (GABA_A_ α1-GABA_A_ α3)/(GABA_A_ α1+GABA_A_ α3). Presynaptic E/I index: (VGLUT1-VGAT)/(VGLUT1+VGAT). Postsynaptic E/I index: (PSD-95-gephyrin)/(PSD-95+gephyrin).

To compare levels of protein expression among the groups we made histograms showing the mean and SEM for each group. All results were plotted normalized to the normal young adult group. To make statistical comparisons between groups we used bootstrapping, a modern resampling statistical method that provides robust estimates of SE and confidence intervals, that is especially useful for animal studies such as ours constrained to smaller sample sizes. We used R to simulate a normally distributed dataset with 1,00,000 points and the same mean and SD as the group being compared. To determine differences between groups, we compared the simulation dataset with average protein expression with each of the other groups. We ran a Monte Carlo simulation which randomly samples from the simulation dataset *N* time, where *N* was the number of animals in each of the other groups (*N*=6 or 8). This simulation was repeated 10,000 times to create the normal distribution expected for the *N* sample sizes. We calculated confidence intervals for the simulated distribution and compared those with the observed means for the other groups. Groups were identified as significantly different (eg, *p* < 0.05) when the observed average expression was either greater or <95% of the simulated distribution and thus outside its confidence interval (Table 1).

**Table 1.** Statistical table

Data point	Data structure	Type of test	95% Confidence interval vs normal	95% Confidence interval vs fluoxetine	95% Confidence interval vs 1 week MD	95% Confidence interval vs fluoxetine + 1 week MD
V1 Ipsi synapsin - Normal	Normal	Bootstrapping + Monte Carlo Simulation	0.8112–1.1888	0.7825–1.2380	1.1441–0.8279	0.7813–0.9824
V1 Ipsi synapsin- fluoxetine	Normal	Bootstrapping + Monte Carlo Simulation	0.8388–1.1612	0.8131–1.2074	1.1275–0.8445	0.7945–0.9692
V1 Ipsi synapsin - 1 week MD	Normal	Bootstrapping + Monte Carlo Simulation	0.8094–1.1906	0.7820–1.2384	1.1457–0.8263	0.7798–0.9839
V1 Ipsi synapsin - fluoxetine + 1 week MD	Normal	Bootstrapping + Monte Carlo Simulation	0.8378–1.1622	0.8126–1.2079	1.1258–0.8462	0.7951–0.9686
V1 Ipsi synaptophysin - Normal	Normal	Bootstrapping + Monte Carlo Simulation	0.8812–1.1188	0.8583–1.1757	0.9164–1.2347	0.9007–1.1095
V1 Ipsi synaptophysin - fluoxetine	Normal	Bootstrapping + Monte Carlo Simulation	0.8957–1.1043	0.8817–1.1523	0.9400–1.2110	0.9152–1.0950
V1 Ipsi synaptophysin - 1 week MD	Normal	Bootstrapping + Monte Carlo Simulation	0.8783–1.1217	0.8604–1.1735	0.9147–1.2364	0.9011–1.1091
V1 Ipsi synaptophysin - fluoxetine + 1 week MD	Normal	Bootstrapping + Monte Carlo Simulation	0.8977–1.1023	0.8782–1.1558	0.9352–1.2159	0.9150–1.0953
V1 Ipsi PSD-95 – Normal	Normal	Bootstrapping + Monte Carlo Simulation	0.6143–1.3857	0.7437–1.2262	1.0125–1.3042	0.9502–1.3776
V1 Ipsi PSD-95 - fluoxetine	Normal	Bootstrapping + Monte Carlo Simulation	0.6678–1.3322	0.7799–1.1900	1.0345–1.2823	0.9780–1.3498
V1 Ipsi PSD-95 - 1 week MD	Normal	Bootstrapping + Monte Carlo Simulation	0.6117–1.3883	0.7469–1.2230	1.0125–1.3043	0.9499–1.3779
V1 Ipsi PSD-95 - fluoxetine + 1 week MD	Normal	Bootstrapping + Monte Carlo Simulation	0.6678–1.3322	0.7799–1.1900	1.0325–1.2843	0.9765–1.3513
V1 Ipsi gephyrin - Normal	Normal	Bootstrapping + Monte Carlo Simulation	0.7124–1.2876	0.7326–1.2570	0.9003–1.1418	0.9795–1.2321
V1 Ipsi gephyrin - fluoxetine	Normal	Bootstrapping + Monte Carlo Simulation	0.7491–1.2509	0.7669–1.2228	0.9169–1.1253	0.9974–1.2142
V1 Ipsi gephyrin - 1 week MD	Normal	Bootstrapping + Monte Carlo Simulation	0.7048–1.2952	0.7297–1.2599	0.9035–1.1386	0.9795–1.2320
V1 Ipsi gephyrin - fluoxetine + 1 week MD	Normal	Bootstrapping + Monte Carlo Simulation	0.7533–1.2467	0.7677–1.2219	0.9182–1.1239	0.9961–1.2155
V1 contra synapsin- normal	Normal	Bootstrapping + Monte Carlo Simulation	0.8105–1.1895	0.7781–1.2424	0.5763–1.1335	0.7372–1.3212
V1 contra synapsin- fluoxetine	Normal	Bootstrapping + Monte Carlo Simulation	0.8384–1.1616	0.8055–1.2150	0.6118–1.0979	0.7764–1.2820
V1 contra synapsin - 1 week MD	Normal	Bootstrapping + Monte Carlo Simulation	0.8138–1.1862	0.7726–1.2478	0.5732–1.1365	0.7322–1.3262
V1 contra synapsin - fluoxetine + 1 week MD	Normal	Bootstrapping + Monte Carlo Simulation	0.8381–1.1619	0.8103–1.2102	0.6112–1.0986	0.7680–1.2904
V1 contra synaptophysin - normal	Normal	Bootstrapping + Monte Carlo Simulation	0.8801–1.1199	0.8473–1.1555	0.7132–1.0231	0.8204–1.0900
V1 contra synaptophysin - fluoxetine	Normal	Bootstrapping + Monte Carlo Simulation	0.8777–1.1223	0.8663–1.1366	0.7315–1.0048	0.8387–1.0717
V1 contra synaptophysin - 1 week MD	Normal	Bootstrapping + Monte Carlo Simulation	0.8959–1.1041	0.8459–1.1569	0.7144–1.0219	0.8202–1.0902
V1 contra synaptophysin - fluoxetine + 1 week MD	Normal	Bootstrapping + Monte Carlo Simulation	0.8930–1.1070	0.8693–1.1336	0.7350–1.0013	0.8389–1.0715
V1 contra VGLUT1 - normal	Normal	Bootstrapping + Monte Carlo Simulation	0.8693–1.1307	0.6128–0.8079	1.0116–1.4828	0.7065–1.0228
V1 contra VGLUT1 - fluoxetine	Normal	Bootstrapping + Monte Carlo Simulation	0.8872–1.1128	0.6250–0.7957	1.0435–1.4509	0.7247–1.0046
V1 contra VGLUT1 - 1 week MD	Normal	Bootstrapping + Monte Carlo Simulation	0.8685–1.1315	0.6107–0.8100	1.0034–1.4910	0.7044–1.0249
V1 contra VGLUT1 - fluoxetine + 1 week MD	Normal	Bootstrapping + Monte Carlo Simulation	0.8876–1.1124	0.6227–0.7980	1.0387–1.4557	0.7228–1.0065
V1 contra VGAT - normal	Normal	Bootstrapping + Monte Carlo Simulation	0.6458–1.3542	0.5777–1.2580	0.6151–1.0278	0.6073–1.3463
V1 contra VGAT - fluoxetine	Normal	Bootstrapping + Monte Carlo Simulation	0.6993–1.3007	0.6330–1.2027	0.6390–1.0039	0.6511–1.3025
V1 contra VGAT - 1 week MD	Normal	Bootstrapping + Monte Carlo Simulation	0.6512–1.3488	0.5808–1.2549	0.6160–1.0269	0.6015–1.3521
V1 contra VGAT - fluoxetine + 1 week MD	Normal	Bootstrapping + Monte Carlo Simulation	0.6956–1.3044	0.6339–1.2019	0.6414–1.0015	0.6515–1.3020
V1 contra PSD-95 - normal	Normal	Bootstrapping + Monte Carlo Simulation	0.6038–1.3962	0.7218–1.1861	0.4462–0.8174	0.9084–2.0180
V1 contra PSD-95 - fluoxetine	Normal	Bootstrapping + Monte Carlo Simulation	0.6683–1.3317	0.7503–1.1575	0.4720–0.7916	0.9719–1.9545
V1 contra PSD-95 - 1 week MD	Normal	Bootstrapping + Monte Carlo Simulation	0.6097–1.3903	0.7204–1.1875	0.4505–0.8131	0.9037–2.0227
V1 contra PSD-95 - fluoxetine + 1 week MD	Normal	Bootstrapping + Monte Carlo Simulation	0.6679–1.3321	0.7493–1.1585	0.4700–0.7937	0.9767–1.9497
V1 contra gephyrin - Normal	Normal	Bootstrapping + Monte Carlo Simulation	0.7050–1.2950	0.7343–1.3432	0.4036–0.7036	0.8690–1.8151
V1 contra gephyrin - fluoxetine	Normal	Bootstrapping + Monte Carlo Simulation	0.7480–1.2520	0.7847–1.2928	0.4257–0.6815	0.9298–1.7543
V1 contra gephyrin - 1 week MD	Normal	Bootstrapping + Monte Carlo Simulation	0.7089–1.2911	0.7444–1.3331	0.4053–0.7019	0.8845–1.7996
V1 contra gephyrin - fluoxetine + 1 week MD	Normal	Bootstrapping + Monte Carlo Simulation	0.7515–1.2485	0.7858–1.2916	0.4304–0.6768	0.9435–1.7406
V1 contra GluN1 - normal	Normal	Bootstrapping + Monte Carlo Simulation	0.8909–1.1092	0.6978–1.0043	1.0128–1.4852	0.6713–0.9632
V1 contra GluN1 - fluoxetine	Normal	Bootstrapping + Monte Carlo Simulation	0.9037–1.0963	0.7187–0.9834	1.0445–1.4536	0.6910–0.9434
V1 contra GluN1 - 1 week MD	Normal	Bootstrapping + Monte Carlo Simulation	0.8910–1.1090	0.6980–1.0042	1.0159–1.4822	0.6696–0.9648
V1 contra GluN1 - fluoxetine + 1 week MD	Normal	Bootstrapping + Monte Carlo Simulation	0.9053–1.0947	0.7206–0.9815	1.0457–1.4523	0.6897–0.9447
V1 contra GluA2 - normal	Normal	Bootstrapping + Monte Carlo Simulation	0.8632–1.1368	0.7766–1.0205	1.0076–1.3460	0.7128–0.9906
V1 contra GluA2 - fluoxetine	Normal	Bootstrapping + Monte Carlo Simulation	0.8824–1.1176	0.7943–1.0028	1.0326–1.3210	0.7128–0.9906
V1 contra GluA2 - 1 week MD	Normal	Bootstrapping + Monte Carlo Simulation	0.8605–1.1395	0.7774–1.0197	1.0017–1.3519	0.7368–0.9667
V1 contra GluA2 - fluoxetine + 1 week MD	Normal	Bootstrapping + Monte Carlo Simulation	0.8790–1.1210	0.7940–1.0030	1.0316–1.3220	0.7310–0.9724
V1 contra GluN2A - normal	Normal	Bootstrapping + Monte Carlo Simulation	0.7104–1.2896	0.6612–1.0471	0.9664–1.5040	0.7161–1.1003
V1 contra GluN2A - fluoxetine	Normal	Bootstrapping + Monte Carlo Simulation	0.7063–1.2937	0.6880–1.0203	1.0035–1.4669	0.7431–1.0733
V1 contra GluN2A - 1 week MD	Normal	Bootstrapping + Monte Carlo Simulation	0.7430–1.2569	0.6628–1.0455	0.9607–1.5097	0.7190–1.0974
V1 contra GluN2A - fluoxetine + 1 week MD	Normal	Bootstrapping + Monte Carlo Simulation	0.7418–1.2582	0.6832–1.0251	1.0056–1.4648	0.7427–1.0737
V1 contra GluN2B - normal	Normal	Bootstrapping + Monte Carlo Simulation	0.7772–1.2228	0.5712–0.8636	0.8007–1.1522	0.6562–0.9201
V1 contra GluN2B - fluoxetine	Normal	Bootstrapping + Monte Carlo Simulation	0.7812–1.2188	0.5881–0.8466	0.8229–1.1300	0.6740–0.9022
V1 contra GluN2B - 1 week MD	Normal	Bootstrapping + Monte Carlo Simulation	0.8074–1.1926	0.5659–0.8688	0.8029–1.1500	0.6584–0.9179
V1 contra GluN2B - fluoxetine + 1 week MD	Normal	Bootstrapping + Monte Carlo Simulation	0.8120–1.1880	0.5862–0.8485	0.8239–1.1289	0.6728–0.9034
V1 contra GABAA3 - normal	Normal	Bootstrapping + Monte Carlo Simulation	0.8712–1.1288	0.7659–1.0577	0.9939–1.3721	0.7512–1.0196
V1 contra GABAA3 - fluoxetine	Normal	Bootstrapping + Monte Carlo Simulation	0.8908–1.1092	0.7856–1.0380	1.0139–1.3520	0.7645–1.0063
V1 contra GABAA3 - 1 week MD	Normal	Bootstrapping + Monte Carlo Simulation	0.8729–1.1271	0.7641–1.0596	0.9921–1.3738	0.7447–1.0261
V1 contra GABAA3 - fluoxetine + 1 week MD	Normal	Bootstrapping + Monte Carlo Simulation	0.8894–1.1106	0.7883–1.0353	1.0207–1.3452	0.7655–1.0053
V1 contra GABAA1 - normal	Normal	Bootstrapping + Monte Carlo Simulation	0.8751–1.1249	0.8854–1.5893	0.7594–1.2798	0.5585–1.9208
V1 contra GABAA1 - fluoxetine	Normal	Bootstrapping + Monte Carlo Simulation	0.8898–1.1102	0.9302–1.5445	0.7971–1.2422	0.6434–1.8359
V1 contra GABAA1 - 1 week MD	Normal	Bootstrapping + Monte Carlo Simulation	0.8713–1.1287	0.8863–1.5883	0.7642–1.2751	0.5339–1.9454
V1 contra GABAA1 - fluoxetine + 1 week MD	Normal	Bootstrapping + Monte Carlo Simulation	0.8883–1.1117	0.9312–1.5435	0.7932–1.2461	0.6465–1.8328
V1 contra GluA2–GluN1 - normal	Normal	Bootstrapping + Monte Carlo Simulation	–0.0675 to 0.0603	–0.0351 to 0.0859	–0.0838 to 0.0295	–0.0379 to 0.0676
V1 contra GluA2–GluN1 – fluoxetine	Normal	Bootstrapping + Monte Carlo Simulation	–0.0595 to 0.0523	–0.0279 to 0.0787	–0.0766 to 0.0223	–0.0304 to 0.0601
V1 contra GluA2–GluN1 - 1 week MD	Normal	Bootstrapping + Monte Carlo Simulation	–0.0675 to 0.0603	–0.0360 to 0.0868	–0.0834 to 0.0291	–0.0370 to 0.0667
V1 contra GluA2–GluN1 - fluoxetine + 1 week MD	Normal	Bootstrapping + Monte Carlo Simulation	–0.0596 to 0.0525	–0.0270 to 0.0778	–0.0774 to 0.0231	–0.0317 to 0.0614
V1 contra GluN2A–GluN2B – Normal	Normal	Bootstrapping + Monte Carlo Simulation	–0.1879 to 0.0107	–0.0659 to 0.0841	–0.0451 to 0.1269	–0.0775 to 0.0616
V1 contra GluN2A–GluN2B – fluoxetine	Normal	Bootstrapping + Monte Carlo Simulation	–0.1755 to –0.0018	–0.0553 to 0.0735	–0.0331–0.1149	–0.0694 to 0.0536
V1 contra GluN2A–GluN2B - 1 week MD	Normal	Bootstrapping + Monte Carlo Simulation	–0.1862 to 0.0090	–0.0679 to 0.0862	–0.0456 to 0.1274	–0.0767 to 0.0608
V1 contra GluN2A–GluN2B - fluoxetine + 1 week MD	Normal	Bootstrapping + Monte Carlo Simulation	–0.1738 to –0.0034	–0.0569 to 0.0752	–0.0347 to 0.1165	–0.0684 to 0.0526
V1 contra GABAA1–GABAA3 - Normal	Normal	Bootstrapping + Monte Carlo Simulation	–0.1582 to 0.0079	–0.0817 to 0.1848	–0.2900 to –0.0325	–0.1058 to 0.1577
V1 contra GABAA1:GABAA3 - fluoxetine	Normal	Bootstrapping + Monte Carlo Simulation	–0.1463 to –0.0039	–0.0619 to 0.1650	–0.2744 to –0.0481	–0.0873 to 0.1392
V1 contra GABAA1:GABAA3 - 1 week MD	Normal	Bootstrapping + Monte Carlo Simulation	–0.1594 to 0.0092	–0.0804 to 0.1835	–0.2919 to –0.0306	–0.1062 to 0.1582
V1 contra GABAA1:GABAA3 - fluoxetine + 1 week MD	Normal	Bootstrapping + Monte Carlo Simulation	–0.1472 to –0.0031	–0.0610 to 0.1641	–0.2729 to –0.0496	–0.0866 to 0.1385
V1 contra Presynaptic E/I - Normal	Normal	Bootstrapping + Monte Carlo Simulation	–0.0981 to 0.1807	–0.2242 to 0.0731	0.0853–0.3517	–0.1710 to 0.1301
V1 contra Presynaptic E/I - fluoxetine	Normal	Bootstrapping + Monte Carlo Simulation	–0.0809 to 0.1635	–0.2033 to 0.0523	0.1032–0.3338	–0.1525 to 0.1116
V1 contra Presynaptic E/I - 1 week MD	Normal	Bootstrapping + Monte Carlo Simulation	–0.0983 to 0.1808	–0.2288 to 0.0777	0.0850–0.3520	–0.1725 to 0.1316
V1 contra Presynaptic E/I - fluoxetine + 1 week MD	Normal	Bootstrapping + Monte Carlo Simulation	–0.0770 to 0.1595	–0.2074 to 0.0563	0.0987–0.3383	–0.1521 to 0.1112
V1 contra Postsynaptic E/I - Normal	Normal	Bootstrapping + Monte Carlo Simulation	–0.1202 to 0.1499	–0.1150 to 0.0745	0.0653–0.3197	–0.0334 to 0.1542
V1 contra Postsynaptic E/I - fluoxetine	Normal	Bootstrapping + Monte Carlo Simulation	–0.0999 to 0.1295	–0.1009 to 0.0604	0.0834–0.3016	–0.0208 to 0.1417
V1 contra Postsynaptic E/I - 1 week MD	Normal	Bootstrapping + Monte Carlo Simulation	–0.1199 to 0.1495	–0.1155 to 0.0750	0.0629–0.3221	–0.0324 to 0.1532
V1 contra Postsynaptic E/I - fluoxetine + 1 week MD	Normal	Bootstrapping + Monte Carlo Simulation	–0.1008 to 0.1304	–0.1021 to 0.0616	0.0818–0.3032	–0.0210 to 0.1418

### Image manipulation

Bands are representative samples taken from different parts of the same gel or different gels. Horizontal and vertical transformations were uniformly applied to size bands appropriately for each figure. A linear adjustment layer was applied uniformly to all bands of each protein, preserving the relative intensities between groups.

## Results

**Fig 1.**
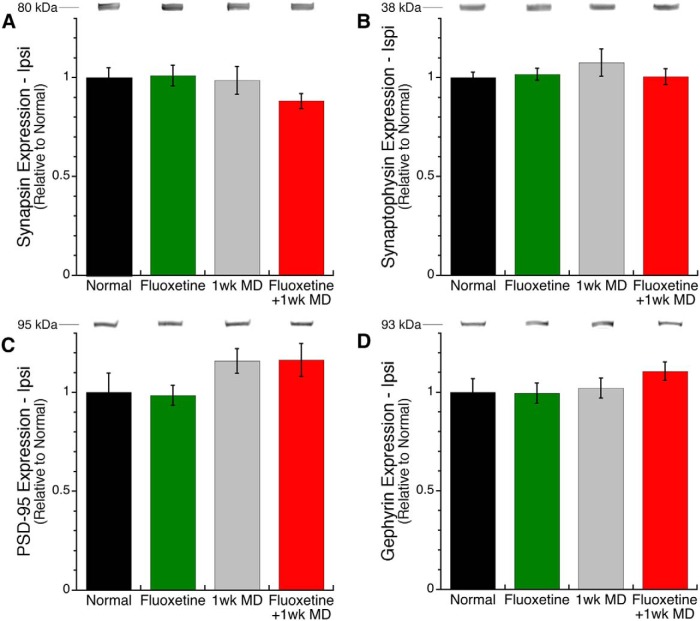
**Presynaptic and postsynaptic proteins in ipsilateral V1.** In V1 ipsilateral to the deprived eye, there was no effect of experimental condition on the expression of synapsin (***A***), synaptophysin (***B***), PSD-95 (***C***), or gephyrin (***D***). *p < 0.05, **p < 0.01, ***p < 0.001, ****p < 0.0001).

We verified that GAPDH was an appropriate loading control by comparing expression of it among the four groups. We found no significant differences from normal demonstrating that GAPDH is an appropriate loading control. We began by examining expression of synapsin, synaptophysin, PSD-95, and gephyrin in V1 ipsilateral to the deprived eye. MD effects are much weaker in the ipsilateral hemisphere (Sawtell et al., 2003) and we did not find any significant differences among the groups for those synaptic proteins (Fig. 1). Thus, all of the following analyses are for V1 contralateral to the deprived eye.

### Presynaptic changes

**Fig 2.**
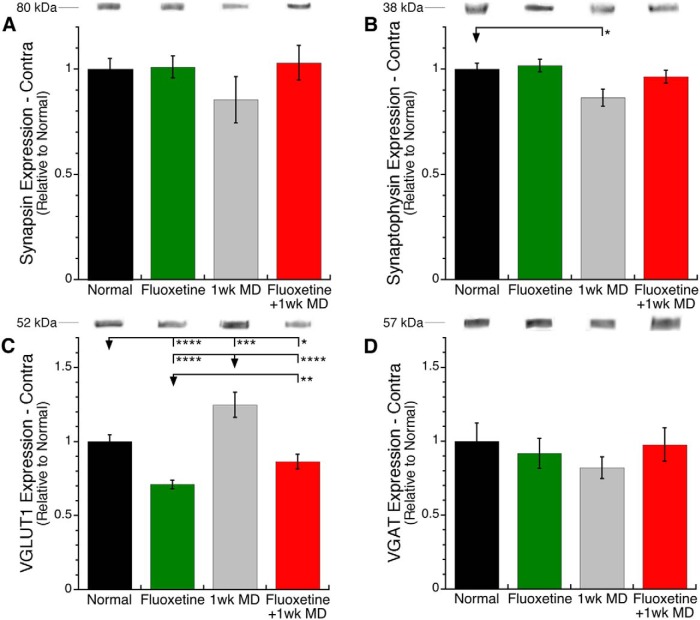
**Presynaptic vesicle cycling and transporter proteins**. In contralateral V1, synapsin (***A***) was not affected by experimental condition. For synaptophysin (***B***) fluoxetine alone had no effect, MD alone caused a loss of expression, but combining fluoxetine with MD prevented the MD-induced loss. For VGLUT1 (***C***) fluoxetine alone or with MD caused a loss of expression, but MD alone increased expression. VGAT (***D***) was not affected by experimental condition. *p < 0.05, **p < 0.01, ***p < 0.001, ****p < 0.0001.

We analyzed how fluoxetine changed the presynaptic environment by quantifying a set of proteins involved in cycling, transport, and loading of glutamatergic and GABAergic vesicles. We compared expression of synapsin, synaptophysin, VGLUT1 and VGAT in V1 of normally reared adult rats, rats given 1 month of fluoxetine, 1 week of MD, or the combination of fluoxetine and MD. We found no differences among the groups for synapsin (n.s.; Fig. 2*A*) or the GABAergic transporter VGAT (n.s.; Fig. 2*D*) and only a modest loss of synaptophysin for the MDed animals (−13%, SEM 4.1%, *p* < 0.05; Fig. 2*B*). The glutamate transporter VGLUT1, however, had more changes. MDed animals had an increase in VGLUT1 (+25%, SEM 8.4%, *p* < 0.001), whereas both groups of fluoxetine treated animals had less VGLUT1 than normal (fluoxetine alone −29%, SEM 3.0%, *p* < 0.0001; fluoxetine+MD −13%, SEM 4.9%, *p* < 0.05; Fig. 2*C*.

### Postsynaptic changes

**Fig 3.**
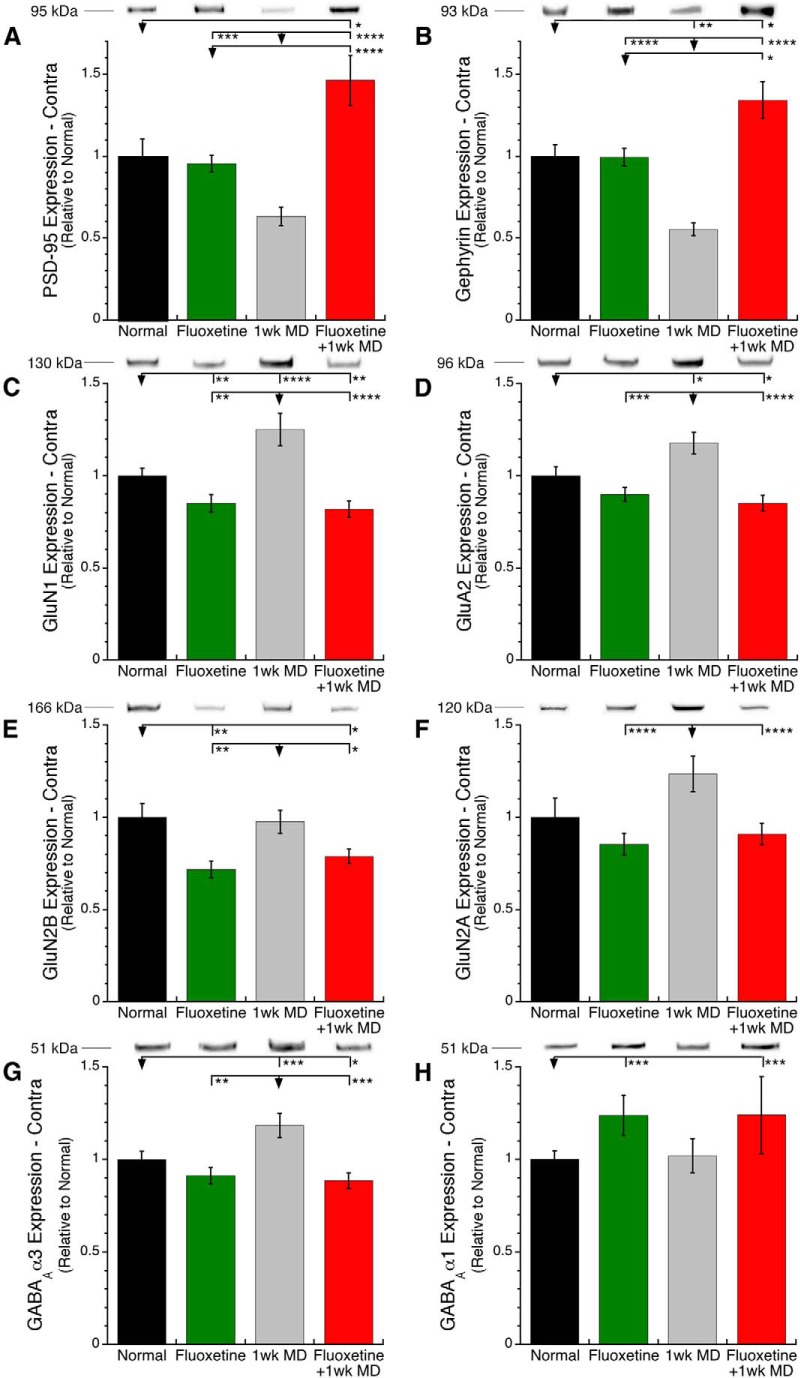
**Postsynaptic receptor scaffolding proteins and subunits.** In contralateral V1, PSD-95 (***A***) and gephyrin (***B***) had a similar pattern of changes: fluoxetine alone had no effect, MD alone caused a loss of expression, but combining fluoxetine with MD prevented the MD-induced loss and caused super-compensation above normal levels. GluN1 (***C***) was reduced by fluoxetine regardless of visual experience, whereas MD alone caused an increase. GluA2 (***D***) was unaffected by fluoxetine alone, MD caused an increase, but combing fluoxetine with MD caused a decrease. GluN2B (***E***) was reduced by fluoxetine regardless of visual experience, whereas MD had no effect. GluN2A (***F***) expression of each experimental group was not different from normal animals, but the MDed group had higher expression than either fluoxetine alone or fluoxetine combined with MD. GABAAα3 (***G***) was unaffected by fluoxetine alone, MD caused an increase, but combing fluoxetine with MD prevented the MD-induced increase. GABAAα1 (***H***) was increased by fluoxetine regardless of visual experience, while MD alone had no effect. *p < 0.05, **p < 0.01, ***p < 0.001, ****p < 0.0001.

Next, we examined how fluoxetine changed the expression of a set of postsynaptic scaffolding proteins and receptor subunits for glutamatergic and GABAergic receptors. Changes among the groups were very similar for PSD-95 and gephyrin. Fluoxetine alone did not change the level of expression relative to normal animals, but MD caused loss of expression (PSD-95 −37%, SEM 5.6%, *p* ∼ 0.06; gephyrin −45%, SEM 4.0%, *p* < 0.01) and fluoxetine combined with MD increased expression (PSD-95 +46%, SEM 15%, *p* < 0.05; gephyrin +34% SEM 11%, *p* < 0.05; Fig. 3*A*,*B*).

The pattern of changes for the receptor subunits was almost opposite to the scaffolding proteins. For the glutamatergic receptor subunits (GluN1, GluA2, GluN2B, GluN2A) fluoxetine alone caused losses for GluN1 and GluN2B (GluN1 −15%, SEM 4.8%, *p* < 0.01; GluN2B −28%, SEM 4.5%, *p* < 0.01) and when combined with MD caused a loss of GluA2, as well as losses for GluN1 and GluN2B (GluA2 −15%, SEM 4.2%, *p* < 0.0001; GluN1 −18%, SEM 4.5%, *p* < 0.0001; GluN2B −21%, SEM 4%, *p* < 0.05; Fig. 3*C*–*F*). However, MD alone caused either an increase (GluN1 +25%, SEM 8.8%, *p* < 0.0001; GluA2 +18%, SEM 5.9%, *p* < 0.05) or no significant change from normal (GluN2B, GluN2A, n.s.). Thus, MD alone caused gains for these glutamatergic subunits that were reduced when MD was combined with fluoxetine. MD also increased GABA_A_α3 (+18%, SEM 6.6%, *p* < 0.001; Fig. 3*G*) but did not change GABA_A_α1 (Fig. 3*H*). In contrast, GABA_A_α1 was increased in both fluoxetine treated groups (fluoxetine alone +24%, SEM 11%, *p* < 0.001; fluoxetine+MD +24%, SEM 20%, *p* < 0.001; Fig. 3*H*).

### Receptor subunit balances

**Fig 4.**
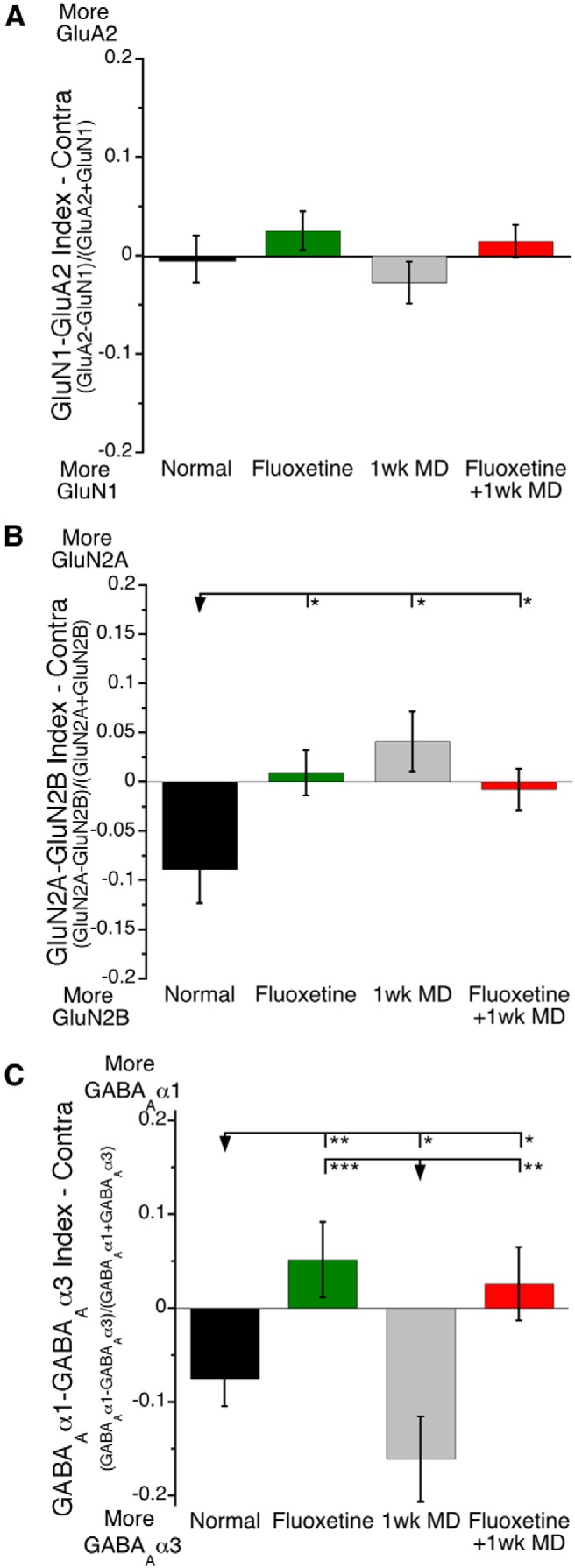
**Postsynaptic receptor subunit balances.** Neither fluoxetine, MD, nor fluoxetine combined with MD affected the relative abundance of GluN1-containing NMDARs and GluA2-containing AMPARs in contralateral V1 (***A***). Fluoxetine shifted the relative abundance of NMDAR subunits in favor of the more mature GluN2A subunit, regardless of visual experience. MD caused a shift in favor of the more immature GluN2B (***B***). Fluoxetine shifted the relative abundance of GABAAR subunits in favor of the more mature α1 subunit, regardless of visual experience. MD caused a shift in favor of the more α3 subunit (***C***). *p < 0.05, **p < 0.01, ***p < 0.001, ****p < 0.0001.

During development, there are a series of maturational shifts in expression of glutamatergic and GABAergic receptor subunits. One of the shifts is the change from NMDAR-dominated silent synapses to AMPAR activated synapses. We studied if fluoxetine created a CP-like state by shifting the GluA2–GluN1 balance in favor of GluN1 but found no changes from the normal adult balance (n.s; Fig. 4*A*). Different results were found when the GluN2A–GluN2B and GABA_A_α1–GABA_A_α3 balances were examined. During normal development there is an increase in GluN2A, shifting the balance from much more GluN2B to slightly in favor of GluN2B in young adult rats (Fig. 4*B*). However, all of the experimental groups changed beyond that level toward even more GluN2A (*p* < 0.05). There were differences, however, in what drove the changes in the GluN2A–GluN2B balance with the fluoxetine groups shift being caused by less GluN2B, whereas the MD shift was caused by more GluN2A. The GABA_A_α1–GABA_A_α3 balance revealed another dissociation among the experimental groups (Fig. 4*C*). Here the MD shift was caused by a 20% increase in GABA_A_α3 (*p* < 0.05), whereas the shift for the fluoxetine groups was caused by a 20% increase in GABA_A_α1 (fluoxetine alone, *p* < 0.01; fluoxetine+MD, *p* < 0.05; Fig. 4*C*). This series of subunit balances unpacks subtle effect of fluoxetine treatment showing that it does not cause a shift to a CP-like state, instead it maintains subunit balances that are like normal adults (GluA2–GluN1) or shifted to more of the mature subunits (GluN2A, GABA_A_α1).

### E/I balances

**Fig 5.**
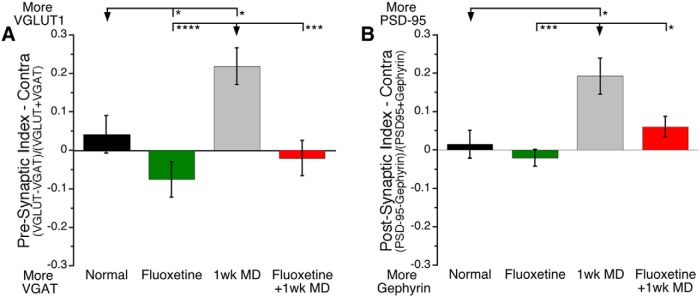
**Presynaptic and Postsynaptic E/I balance.** Presynaptic Index in contralateral V1 (***A***): (VGLUT1−VGAT)/(VGLUT1+VGAT). Postsynaptic Index in contralateral V1 (***B***): (PSD-95−gephyrin)/(PSD-95+gephyrin). We found strikingly similar patters in the presynaptic and postsynaptic indexes of E/I synapses. Fluoxetine caused a slight shift toward inhibition in the presynaptic index and had no effect on the postsynaptic index. MD caused a strong shift to excitatory markers. Combining fluoxetine and MD kept the balance at normal levels. *p < 0.05, **p < 0.01, ***p < 0.001, ****p < 0.0001.

The final analyses examined presynaptic and postsynaptic proteins that regulate the E/I balance. First, we calculated a presynaptic E/I balance using the vesicular transporters VGLUT1 and VGAT. MD caused a large shift toward VGLUT1 (*p* < 0.05; Fig. 5*A*) but when combined with fluoxetine there was no change in the presynaptic E/I balance. The same pattern was seen on the postsynaptic side, here MD also caused a large shift toward the excitatory side (more PSD-95; *p* < 0.05; Fig. 5*B*) but when MD was paired with fluoxetine there was no change from the normal adult E/I balance.

## Discussion

In this study, we quantified the effect of fluoxetine treatment on 12 glutamatergic and GABAergic markers linked with visual experience-dependent plasticity in V1. Fluoxetine caused a pattern of change in those markers that provides new insights into how this drug affects plasticity in adult V1. We compared normal adult rats with ones treated with either fluoxetine alone, MD, or fluoxetine paired with MD. The main findings are that fluoxetine treatment in adult rats does not shift these markers to a younger pattern but instead rebalances MD driven glutamatergic gain and promotes a novel synaptic environment.

In this study, we used Western blotting to quantify the effects of fluoxetine treatment on a collection of synaptic proteins in adult V1. A strength of this approach is that a large number of synaptic proteins were analyzed. Western blotting, however, does not provide laminar or cell-specific information that is needed to identify the neural circuits in V1 affected by fluoxetine. Future neuroanatomical studies are needed to address that question and those studies may be guided by the current results.

### Fluoxetine does not recreate a younger synaptic environment

An appealing hypothesis about drug treatments, such as fluoxetine, is that they reinstate ocular dominance plasticity in adult V1 by changing the synaptic environment to a CP-like state. During the CP, there are increases in amount of proteins and shifts in balances between functional pairs of synaptic proteins. Our results do not support the idea that fluoxetine in adult rats dials back synaptic age. For example, we found that fluoxetine combined with MD caused greater expression of PSD-95 and gephyrin. These levels were higher than found during the CP (Pinto et al., 2015) and are consistent with a spike in PSD-95 that ends the CP (Huang et al., 2015). Furthermore, fluoxetine alone did not reduce expression of either scaffolding protein and only MD caused a loss of PSD-95 and gephyrin. The modest losses for VGLUT1 and receptor subunits caused by fluoxetine suggest a shift to a more immature stage, but the balances among the subunits do not support that conclusion. If a younger synaptic environment was recreated then it should favor NMDA over AMPA receptors (Wu et al., 1996), GluN2B over GluN2A (Carmignoto and Vicini, 1992; Flint et al., 1997; Stocca and Vicini, 1998) and GABA_A_α3 over GABA_A_α1 (Laurie et al., 1992). Instead, the NMDAR to AMPAR ratios were balanced for both fluoxetine groups (GluN1∼GluA2), whereas subunit balances jumped past age-matched adults toward even more of the mature subunits (GluN2A, GABA_A_α1). Finally, E/I balances for presynaptic and postsynaptic markers were similar to the normal adults in both fluoxetine groups. Together these findings illustrate that fluoxetine treatment did not simply recreate a CP-like synaptic environment in V1.

It is important to note that we examined synaptic proteins after 1 month of fluoxetine treatment and 1week of MD. We know from previous studies (Williams et al., 2015) that there are dynamic changes in synaptic proteins during a period of MD and it seems reasonable to propose that fluoxetine treatment may cause similarly dynamic changes. Thus, the findings here provide a snapshot of long-term effects of fluoxetine treatment. It will be important for future studies to probe other time points to understand the full landscape of synaptic changes and how transient changes caused by fluoxetine (Vetencourt et al., 2011) impact long-term plasticity in V1.

### Fluoxetine triggers a novel synaptic environment in adult V1

The original study showing that fluoxetine reinstates ocular dominance plasticity also found improvement of visual function, reduced intracortical inhibition, and increased BDNF expression (Vetencourt et al., 2008). All of those changes occurred without significantly altering neuronal responsiveness or orientation selectivity in V1. Here we found normal presynaptic and postsynaptic E/I balances, and adult levels of GABA_A_α1 that could support normal responsiveness and orientation selectivity. A previous study of gene expression found reduced VGAT after fluoxetine treatment but no changes in other genes associated with E/I mechanisms (Tiraboschi et al., 2013). We, however, did not find that fluoxetine caused a loss of VGAT protein expression. Some forms of GABAergic plasticity involve changes in VGAT protein expression associated with the amount of neurotransmitter in vesicles (Hartman et al., 2006), and the lack of change in VGAT makes it unlikely that fluoxetine altered this type of plasticity.

A recent proteomic analysis found that fluoxetine caused alterations in cytoskeleton organization, endocytosis, molecular transport, intracellular signaling, redox cellular state, metabolism, and protein degradation (Ruiz-Perera et al., 2015). Those changes included proteins that regulate AMPAR and GABA_A_R, and may affect the E/I balance. Nonetheless, our quantification of synaptic proteins, along with the gene and proteomic studies, show that fluoxetine affects mechanisms that regulate experience-dependent plasticity.

The GluN2A–GluN2B and GABA_A_α1–GABA_A_α3 balances were both affected by fluoxetine and importantly the GABA_A_ balance differentiated fluoxetine treatments from the effects of MD. The changes in these functional pairs of glutamatergic and GABAergic receptor subunits suggest that fluoxetine creates a novel synaptic environment in adult V1. An environment that is dominated by GluN2A and GABA_A_α1 but also has balanced levels of presynaptic and postsynaptic E/I markers. Both GluN2A and GABA_A_α1 subunits are described as mature components because they gradually increase during development and affect plasticity. For example, the developmental shift from GluN2B to more GluN2A speeds up receptor kinetics (Cull-Candy et al., 2001), changes cellular signaling (Kim et al., 2005; Wang et al., 2011), relieves GluN2B negative regulation of AMPARs (Hall and Ghosh, 2008), and controls metaplasticity in V1 (Philpot et al., 2007). GABA_A_α1 is necessary for normal development of orientation tuning (Fagiolini et al., 2004) and gamma rhythms (Cardin et al., 2009; Sohal et al., 2009). The prevalence of GABA_A_α1-positive synapses on pyramidal cell bodies makes them important components in GABAergic regulation of experience-dependent plasticity (Hensch, 2005; Griffen and Maffei, 2014). The different roles of these subunits in experience-dependent plasticity suggests that fluoxetine creates a unique synaptic environment in adult V1 that can support both GluN2A-dependent metaplasticity and GABAergic regulation of ocular dominance plasticity.

### How might fluoxetine trigger adult plasticity?

Reduced intracortical GABA and GABAergic transmission have been found after fluoxetine treatment (Vetencourt et al., 2008; Baroncelli et al., 2011). In contrast, we found a small increase in GABA_A_α1 expression and no loss of GABA_A_α3 or VGAT in rats treated with fluoxetine. Previous studies have shown that fluoxetine positively modulates GABA_A_ receptors and one way is by increasing receptor sensitivity to small amounts of GABA (Robinson, 2002). The α1 subunit is one of the subtypes that confer that increased sensitivity and perhaps more GABA_A_α1 expression modulates GABAergic drive when the amount of neurotransmitter is reduced by fluoxetine. Interestingly, during the CP a brief exposure to vision after deprivation causes a rapid rebound potentiation in miniature IPSCs (mIPSCs) that is correlated with an increase in GABA_A_Rs (Gao et al., 2014). Perhaps the increase in GABA_A_α1 expression found here supports a similar potentiation of mIPSCs and because GABA_A_α1-containing synapses form a key part of the neural circuitry involved in ocular dominance plasticity (Hensch, 2005) fluoxetine may drive a compensatory mechanisms where sensitized GABA_A_Rs enhance adult plasticity.

We also found that fluoxetine caused changes to glutamatergic receptor subunit expression. Fluoxetine is known to inhibit NMDA receptors and may provide neuroprotective effects by regulating glutamatergic involvement in excitotoxicity (Szasz et al., 2007). We found that fluoxetine paired with MD ameliorated glutamatergic gain driven by MD alone, suggesting that one of fluoxetine's effects in adult V1 may be neuroprotective. Fluoxetine acts by inhibiting GluN2B-containing NMDARs (Kiss et al., 2012) and that may trigger increases in both BDNF and AMPARs. GluN2B-mediated signaling inhibits AMPAR trafficking and the amount of GluA2-containing AMPARs (Kim et al., 2005; Derkach et al., 2007; Hall et al., 2007; Hall and Ghosh, 2008) through unique cellular processes that include Ras/ERK, αCamKII, and mTor pathways (Kim et al., 2005; Wang et al., 2011). One way that fluoxetine could affect adult ocular dominance plasticity is if the loss of GluN2B changes the length of GluN2B-mediated Ras/ERK activation (Kim et al., 2005) thereby increasing insertion of AMPAR into synapses and supporting long-term potentiation (LTP). ERK activation is necessary for ocular dominance plasticity in developing V1 (Di Cristo et al., 2001) and fluoxetine in adult animals may enhance ERK-dependent plasticity through the loss of GluN2B.

During the CP ocular dominance plasticity reflects the depression of deprived eye responses but in adults MD leads to enhancement of open-eye responses in V1 (Sawtell et al., 2003). That adult plasticity is dependent on activation of NMDARs and may use Hebbian [LTP, long-term depression (LTD), spike time-dependent plasticity], homeostatic, or metaplasticity (synaptic modification threshold) mechanisms (for review, see Hofer et al., 2006). Visual experience-driven changes to LTP and LTD during the CP depend on GluN2A and previous studies have identified shifts in the GluN2A–GluN2B balance as the mechanism underlying an adjustable synaptic modification threshold in V1 (Philpot et al., 2007). Perhaps the shift to balanced GluN2A–GluN2B expression after fluoxetine treatment is an indication that metaplasticity plays a dominant role in fluoxetine driven adult plasticity. Interestingly, in auditory cortex fluoxetine reduces the potential for LTP (Dringenberg et al., 2014) raising the possibility that the effects of fluoxetine might not be uniform across the cortex.

Fluoxetine could also trigger events similar to those promoted by other NMDAR antagonists that cause a transient burst of glutamate, followed by BDNF release and synapse formation (Duman and Aghajanian, 2014). BDNF plays a key role in fluoxetine’s reactivation of plasticity (Castrén and Rantamäki, 2010) suggesting that a fluoxetine induced loss of GluN2B signaling may enhance BDNF and AMPAR involvement in experience-dependent plasticity in adult V1. Thus, fluoxetine appears to enhance glutamatergic and GABAergic mechanisms that support experience-dependent plasticity in adult V1.

### Implications for other therapies

A variety of other methods are being explored to promote adult recovery from persistent amblyopia, such as dark rearing in animals (He et al., 2006, 2007; Montey and Quinlan, 2011; Duffy and Mitchell, 2013), manipulation of the brakes on plasticity including PirB (Bochner et al., 2014) and chondroitin sulphate proteoglycans (Pizzorusso et al., 2002; Morishita et al., 2010; Bukhari et al., 2015), environmental enrichment (Sale et al., 2007), patterned visual stimulation (Montey et al., 2013), or perceptual learning (Levi and Li, 2009; Baroncelli et al., 2011; Bonaccorsi et al., 2014; Tsirlin et al., 2015). All of these appear to reactivate a certain degree of plasticity that can support ocular dominance plasticity and even visual recovery. The cellular mechanisms typically include LTP of cortical synapses, and although some molecular changes have been identified (He et al., 2006), the full extent has yet to be explored. Do these other techniques mimic the novel pattern of fluoxetine driven glutamatergic and GABAergic changes or do they create different synaptic environments? These are important questions to answer to determine whether these adult manipulations activate one or many different forms of experience-dependent plasticity in V1.

Future studies will need to determine the long-term consequences of fluoxetine-induced changes in adult V1. It is not clear whether stopping drug treatment will allow the synaptic environment to shift back to a normal adult state. In addition, if not what effects that new synaptic environment may have on neural function in the long-term. Finally, it will be important to determine how much of these effects are driven by the increase in serotonin, as opposed to unique effects of fluoxetine. Each of these are important questions to answer that well help to understand plasticity in adult V1 and translate that knowledge into effective treatments for persistent amblyopia.

## Synthesis

The decision was a result of the Reviewing Editor Marlene Bartos and the peer reviewers coming together and discussing their recommendations until a consensus was reached. A fact-based synthesis statement explaining their decision and outlining what is needed to prepare a revision is listed below. The following reviewers agreed to reveal their identity: Hans Dringenberg, Nicoletta Berardi

Both reviewers were very excited about the novelty of data presented in the manuscript. 'This manuscript describes interesting and novel findings regarding the effect of fluoxetine on V1 plasticity and synaptic functioning. There is considerable interest in using fluoxetine to promote plasticity of cortical circuits, an effect that is thought to involve up-regulation of synaptic proteins that predominate during the earlier, critical/sensitive periods of cortical maturation. Here, the authors directly test this hypothesis in a comprehensive and detailed manner. The results are surprising and challenge the notion that fluoxetine reinstates critical period-like plasticity in V1.' 'The data are clear and new. Experimental design appears appropriate. Statistical analysis is appropriate.

However, a main weakness was related to the unspecificity of homogenates. They prevent a direct link between changes on the protein level and specific cell types. Moreover, antibody labelling against the various molecules found to be affected by fluoxetine would add great significance, because it would show whether the effects found are cell-specific. The lack of physiological data was viewed as a diminishing factor. If electrophysiological data (see below) cannot be provided by the authors, than they should at least discuss the possible functional consequences (e.g. IPSP, mIPSCs) of the changes in protein expression.

Reviewer 1:

Specific comments:

1. p. 4, line 57: define "EPSC" on first use

2. p. 4, line 66: "...of the influence of fluoxetine treatment of adult plasticity..." should be "...fluoxetine treatment ON adult plasticity"

3. p. 4, lines 69-70: "To address this, we treated animals with fluoxetine AND quantified a set of glutamatergic and GABAergic synaptic proteins to (asses) ASSESS if they changed to a CP-like state. (When) WE then..."

4. p. 5, sentence "But when combined with MD, fluoxetine treatment normalized the glutamatergic shift found in adult MD rats.": Avoid using "But" at the beginning of a new sentence. Also, avoid vague language: what is a "glutamatergic shift"? You probably mean "an increase in glutamatergic strength of synapses in V1 representing the non-deprived eye"?

5. p. 6, line 83: be consistent in use of abbreviations, MD for monocular deprivation, not MDep as is used here.

6. Results: The authors collected bilateral samples of V1 tissue. However, in the Results section, it is never specified if data are for the V1 ipsilateral or contralateral to the deprived eye. I assume it is always contralateral, but this needs to be stated. Also, what happened in ipsilateral V1?

7. The authors need to clarify whether their samples included both the monocular and binocular zones of V1. In the Discussion, it should be mentioned that these zones differ in terms of synaptic inputs and plasticity mechanisms?

8. p. 11, line 191-192 and elsewhere: When reporting means for decreases in expression, use negative numbers e.g., less VGLUT1 should be -29%.

9. p. 14, first sentence: check grammar; also, first part of sentence not necessary.

10. p. 16, lines 290-291: "A gene study found reduced VGAT after fluoxetine treatment but no changes in other genes associated with E/I mechanisms (Tiraboschi et al., 2013)." Re-word this sentence; what is a "gene study"? Tiraboschi et al. examined gene expression, not genes.

11. p. 19, last sentence of top paragraph, "Thus, fluoxetine appears to set up an array of glutamatergic mechanisms in adult V1 that are known to support experience-dependent plasticity (Cooke and Bear, 2014)." 

This seems very much at odds with some of the experimental results and previous discussion, specifically the failure of fluoxetine to create a CP-like synaptic environment and the increase (absolute and/or relative) in receptor subunits that indicate a more mature synaptic state. To reconcile these discrepancies, it would be important to discuss the difference (as shown in prior work) in synaptic mechanisms mediating OD plasticity in juvenile and adult animals. Given that these mechanisms differ, and that fluoxetine does not re-create CP-like synaptic environments, it seems that it may act on those mechanisms that are particularly important for OD shifts in adulthood. This possibility and its implications need to be addressed.

12. p. 25, line 456: This reference is incomplete and should be: Dringenberg, H.C., Branfield Day, L.R., and Choi, D.H. (2014) Chronic fluoxetine treatment suppresses plasticity (long-term potentiation, LTP) in the mature rodent primary auditory cortex in vivo. Neural Plasticity, vol. 2014, Article ID 571285, 9 pages, 2014. doi:10.1155/2014/571285.

13.p. 27, line 495: incomplete reference

14. p. 30, line 544: incomplete reference

Reviewer 2:

Major point: analysis of homogenates prevents from linking the changes found with specific cell populations. An immuno for the molecules found to be affected by fluoxetine would add great significance, because it would show whether the effects found are cell-specific.

The lack of physiological date (e.g., IPSP, minis,....) diminishes the relevance of the paper. At least, in the discussion, an stronger effort to link the molecular effects with functional properties should be done.

Minor points:

In the Method section it is stated that samples of both V1 cortices were taken, the ipsilateral and the contralateral to the deprived eye. In figures, it seems that only data for the contra cortex are reported, although this is never stated, not even in the captions. Could the authors clarify this?

Line 41-42: a small typo, "During the CP, experience-dependent plasticity is driven by visually evoked responses that dependent upon....."
